# Antibiotic Prescribing Patterns in Ghana, Uganda, Zambia and Tanzania Hospitals: Results from the Global Point Prevalence Survey (G-PPS) on Antimicrobial Use and Stewardship Interventions Implemented

**DOI:** 10.3390/antibiotics10091122

**Published:** 2021-09-17

**Authors:** Nikki D’Arcy, Diane Ashiru-Oredope, Omotayo Olaoye, Daniel Afriyie, Zainab Akello, Daniel Ankrah, Derrick Mawuena Asima, David C. Banda, Scott Barrett, Claire Brandish, Joseph Brayson, Peter Benedict, Cornelius C. Dodoo, Frances Garraghan, Josephyn Hoyelah, Yogini Jani, Freddy Eric Kitutu, Ismail Musoke Kizito, Appiah-Korang Labi, Mariyam Mirfenderesky, Sudaxshina Murdan, Caoimhe Murray, Noah Obeng-Nkrumah, William J’Pathim Olum, Japheth Awuletey Opintan, Edwin Panford-Quainoo, Ines Pauwels, Israel Sefah, Jacqueline Sneddon, Anja St. Clair Jones, Ann Versporten

**Affiliations:** 1 Commonwealth Partnerships Programme on Antimicrobial Stewardship, Commonwealth Pharmacists Association, London E1W 1AW, UK; nikki.darcy@commonwealthpharmacy.org (N.D.); omotayo.olaoye@commonwealthpharmacy.org (O.O.); 2 Pharmacy Department, Ghana Police Hospital, Accra P.O. Box CT104, Ghana; dspdan77@yahoo.com; 3 Laro Division, Gulu Regional Referral Hospital, Gulu P.O. Box 166, Uganda; zainake99@gmail.com; 4 Korle-Bu Teaching Hospital, Accra P.O. Box 77, Ghana; d.ankrah@kbth.gov.gh; 5 LEKMA Hospital, P.M. B Teshie, Nungua, Accra P.O. Box CT104, Ghana; mawuenaasima@gmail.com; 6 Department of Pharmacy, University Teaching Hospital, Lusaka P.O. Box 50001, Zambia; banda.chimbi@gmail.com; 7 Pharmacy Department, North Tyneside Hospital, Northumbria Healthcare NHS Foundation Trust, Rake Lane, North Shields NE29 8NH, UK; Scott.Barrett@northumbria-healthcare.nhs.uk (S.B.); Joseph.Brayson@northumbria-healthcare.nhs.uk (J.B.); 8 Buckinghamshire Healthcare NHS Trust, Amersham HP7 0JD, UK; claire.brandish@nhs.net; 9 Kilimanjaro Christian Medical Centre, Moshi P.O. Box 3010, Tanzania; peterbenedict55@gmail.com; 10 School of Pharmacy, University of Health and Allied Sciences, PMB 31, Ho, Ghana; cdodoo@uhas.edu.gh; 11 Manchester University NHS Foundation Trust, Oxford Road, Manchester M13 9WL, UK; frances.garraghan@mft.nhs.uk; 12 St. Mary’s Hospital Lacor, Gulu P.O. Box 180, Uganda; oyella.josephine@lacorhospital.org; 13 Centre for Medicines Optimisation Research and Education, University College London Hospitals NHS Foundation Trust, 250 Euston Road, London NW1 2PG, UK; yogini.jani@nhs.net; 14 Sustainable Pharmaceutical Systems (SPS) Unit, Pharmacy Department, School of Health Sciences, Makerere University, Kampala P.O. Box 7062, Uganda; freddy.kitutu@mak.ac.ug; 15 Entebbe Regional Referral Hospital, Entebbe P.O. Box 29, Uganda; ismailkizito11@gmail.com; 16 Medical Microbiology Department, University of Ghana Medical School, Accra P.O. Box GP 4236, Ghana; aklabi@ug.edu.gh (A.-K.L.); jaopintan@ug.edu.gh (J.A.O.); 17 North Middlesex University Hospital NHS Trust, London N18 1QX, UK; mariyam.mirfenderesky@nhs.net; 18 UCL School of Pharmacy, University College London, 29-39 Brunswick Square, London WC1N 1AX, UK; s.murdan@ucl.ac.uk; 19 St. Francis Hospital, Katete Private Bag 11, Zambia; caoimhe.murray@nhs.net; 20 Department of Medical Laboratory Sciences, University of Ghana School of Biomedical and Allied Health Sciences, Accra P.O. Box LG 25, Ghana; nobeng-nkrumah@ug.edu.gh; 21 Jinja Regional Referral Hospital, Jinja P.O. Box 43, Uganda; pjathim@gmail.com; 22 Liverpool School of Tropical Medicine, Liverpool L3 5QA, UK; 248964@lstmed.ac.uk; 23 Laboratory of Medical Microbiology, Vaccine & Infectious Disease Institute (VAXINFECTIO), Faculty of Medicine and Health Sciences, University of Antwerp, Universiteitsplein 1, 2610 Antwerp, Belgium; Ines.Pauwels@uantwerpen.be (I.P.); ann.versporten@uantwerpen.be (A.V.); 24 Department of Pharmacy, Keta Municipal Hospital, Keta-Dzelukope P.O. Box WT82, Ghana; isefah@uhas.edu.gh; 25 Scottish Antimicrobial Prescribing Group, Healthcare Improvement Scotland, Delta House, 50 West Nile Street, Glasgow G1 2NP, UK; jacqueline.sneddon@nhs.scot; 26 Brighton and Sussex University Hospitals NHS Trust, Brighton BN1 9PX, UK; anja.st.clair-jones@nhs.net

**Keywords:** antimicrobial resistance, global-pps, antimicrobial surveillance, antibiotics, antimicrobials, antimicrobial stewardship

## Abstract

Antimicrobial resistance (AMR) remains an important global public health issue with antimicrobial misuse and overuse being one of the main drivers. The Global Point Prevalence Survey (G-PPS) of Antimicrobial Consumption and Resistance assesses the prevalence and the quality of antimicrobial prescriptions across hospitals globally. G-PPS was carried out at 17 hospitals across Ghana, Uganda, Zambia and Tanzania. The overall prevalence of antimicrobial use was 50% (30–57%), with most antibiotics prescribed belonging to the WHO ‘Access’ and ‘Watch’ categories. No ‘Reserve’ category of antibiotics was prescribed across the study sites while antimicrobials belonging to the ‘Not Recommended’ group were prescribed infrequently. Antimicrobials were most often prescribed for prophylaxis for obstetric or gynaecological surgery, making up between 12 and 18% of total prescriptions across all countries. The most prescribed therapeutic subgroup of antimicrobials was ‘Antibacterials for systemic use’. As a result of the programme, PPS data are now readily available for the first time in the hospitals, strengthening the global commitment to improved antimicrobial surveillance. Antimicrobial stewardship interventions developed included the formation of AMS committees, the provision of training and the preparation of new AMS guidelines. Other common interventions included the presentation of findings to clinicians for increased awareness, and the promotion of a multi-disciplinary approach to successful AMS programmes. Repeat PPS would be necessary to continually monitor the impact of interventions implemented. Broader participation is also encouraged to strengthen the evidence base.

## 1. Introduction

Antimicrobial resistance (AMR) is a global, public, and individual health challenge affecting the delivery of safe, effective healthcare in all settings and all countries. The ability of microorganisms to become resistant to the effect of antimicrobials is an inevitable evolutionary process; however, misuse and over-use of antimicrobial agents hastens the development and spread [1,2].

AMR leads to increased mortality rates [3] and duration and cost of patient care [1]. This is of particular concern in low- and middle-income countries (LMICs) due to the reduced availability of appropriate equipment and/or appropriate diagnostic tools, as well as challenges with access to quality antimicrobials [4,5,6,7]. The rise in incidence of AMR has led to an increased global focus on antimicrobial stewardship (AMS). Surveillance of antimicrobial use and resistance are core to all AMS activities and understanding how antimicrobials are used allows for a review of current practices and highlights areas for improvement. Point prevalence surveys (PPSs) are a widely recognised surveillance method requiring limited resources to collect information on antimicrobial prescribing practices and other relevant factors in hospitalized patients [8,9,10,11].

PPS are a key resource when planning and supporting national and local stewardship interventions in a range of settings, offering a standardised method for comparing data on antimicrobial use across hospitals and countries. The Global Point Prevalence Survey of Antimicrobial Consumption and Resistance (G-PPS; www.global-pps.com/, accessed on 15 May 2021) aims to assess the global prevalence of antimicrobial prescribing and resistance, with an emphasis on countries with low resources, support, and expertise and supports antimicrobial stewardship programs in order to enhance appropriate antimicrobial prescribing [12].

Alongside the evaluation of antimicrobial prescribing practices in hospitals, PPS can identify targets for quality improvement of antimicrobial prescribing and implement and monitor the impact of interventions through repeated surveys. One of the main aims in strengthening global AMS is to reduce the use of antimicrobials that are in the World Health Organization’s (WHO) ‘Watch’ and ‘Reserve categories’ and ‘Not Recommended’ group of their AWaRe framework [13,14]. The framework recommends preferred antimicrobial choice for treating common infections—the ‘Access’ category, based on consideration of benefits versus risks to patients and the potential for resistance. An additional classification—‘Not recommended’ was added to the framework more recently to include fixed-dose combinations of broad-spectrum antibiotics for which use is not evidence-based [15]. Similarly, the Anatomic Therapeutic Chemical (ATC) classification categorizes drugs active substances into different groups and subgroups according to their therapeutic, pharmacological and chemical properties. WHO endorses the ATC classification as the standard for drug utilization monitoring and research [16].

The Commonwealth Partnerships for Antimicrobial Stewardship programme (CwPAMS), managed by the Commonwealth Pharmacists Association (CPA) and Tropical Health and Education Trust (THET), is a health partnerships programme funded by the UK Official Development Assistance (ODA), through the Department of Health and Social Care’s Fleming Fund to address AMR globally [17]. The aim of the programme is to enhance the implementation of protocols and evidenced-based decision making to support antimicrobial prescribing and the capacity for surveillance of antimicrobial consumption and stewardship.

The CwPAMS programme included 12 partnerships between UK health institutions and counterparts in four African Commonwealth countries: Ghana (GH), Uganda (UG), Zambia (ZM), and Tanzania (TZ). The partnerships consisted of volunteer health workers and experts from the five countries who shared skills and knowledge to co-develop strategies to address AMR and AMS. As part of the fulfillment of the aims of the partnership, a Global Point Prevalence Study was used to obtain baseline data and measure the impact of the implementation of AMS programmes across partnership countries.

This paper aims to compare national data on antimicrobial use obtained from 12 hospitals across four countries (Ghana (6), Uganda (4), Zambia (1) and Tanzania (1)) and identify target points for improvement. As part of an additional collaboration, PPS data from a further four hospitals in Ghana and one additional hospital in Zambia (which collected data using the Global PPS platform during the same period as the CwPAMS programme), are also included in the study.

## 2. Results

### 2.1. Characteristics of Included Hospitals and Eligible Patients

A total of 4376 patients were included in the survey, with 2169 (50%) treated with antimicrobials across the four countries. From the total number of treated patients, 1366 (63%) were from 10 hospitals in Ghana, 386 (17.8%) from four hospitals in Uganda, 238 (11%) from two hospitals in Zambia, and 179 (8%) from one hospital in Tanzania (Table 1). Of the 17 hospitals included, 3 were identified as primary care, 6 as secondary care and 8 as tertiary care. Additionally, eight hospitals were classed as teaching hospitals, three in both Uganda and Ghana and one in both Zambia and Tanzania.

Table 1 summarises the general characteristics of the patients surveyed across the four countries. More adults were included across all sites than children or neonates. A greater proportion of children were included in Uganda (35%) than the other three countries (Ghana (19%), Tanzania (16%), Zambia (15%). Activities of the wards where patients were surveyed were relatively uniform across the countries, with patients on intensive care wards (IC) making up the smallest proportion of those surveyed (4–20%).

### 2.2. Prevalence of Antimicrobial Use

The prevalence of antimicrobial use (AMU) was 50% from the 17 sites across the four countries (Table 2). The proportion of patients treated with antimicrobials at the time of the survey was highest in Zambia (57%), followed by Ghana (55%) and Uganda (45%), and was lowest in Tanzania (30%).

There was a total of 3838 prescriptions recorded in the survey, 2435 (63%) of which were reported from hospitals in Ghana, 710 (18.5%) from Uganda, 402 (10.5%) from Zambia, and 290 (7.5%) from Tanzania.

**Figure 1:**
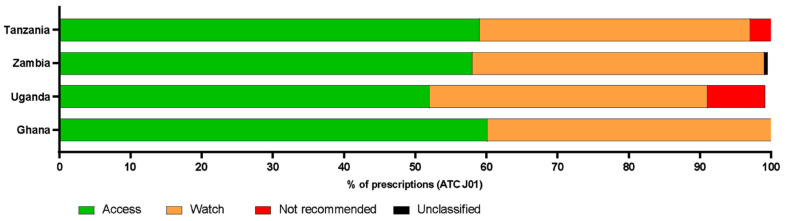
The proportion of antibiotics in the ACT J01 group prescribed in each of four countries.

Of these prescriptions, those in the ATC J01 category (antibacterials for systemic use) were further classified into each of the WHO AWaRe categories (Figure 1). There was no reported use (0%) of the Reserve category of antibiotics in any of the four countries.

Antibiotics in the WHO access category were prescribed more frequently than those in other categories across all countries and were prescribed at similar rates, accounting for 60% of the total of ATC J01 antibiotics prescribed in Ghana (1212/2071), 52% in Uganda (309/592), 58% in Zambia (201/347), and 59% in Tanzania (170/288) (Figure 1). Antibiotics in the Watch category were the second most frequently prescribed, again with proportions being similar for all countries (Ghana 42%; *n* = 851, Uganda 39%; *n* = 232, Zambia 41% *n* = 143, Tanzania 38%; *n* = 109). There was no reported use (0%) of the Reserve category antibiotics in any of the four countries.

‘Not Recommended’ antibiotic combinations were prescribed in Uganda, Tanzania, and Ghana. Ugandan hospitals had the highest prescription rate of these drugs (8%; 48/592) with 47 prescriptions of Ampiclox^®®^ (ampicillin and cloxacillin) (7% of total prescriptions) and one of ciprofloxacin and metronidazole. In Tanzania, Ampiclox^®®^ was prescribed on 9 occasions (3% of total prescriptions). In Ghana, the “Not Recommended” group of antibiotics made up 0.2% of antibiotic prescriptions (4/1212) with ceftriaxone/beta-lactamase inhibitor being prescribed twice, ceftriaxone in combination with another unspecified antibiotic prescribed once and penicillin in combination with another, unspecified antibacterial also prescribed once. Unclassified antibiotics were prescribed very infrequently in all countries.

The proportion of antimicrobials used, as grouped according to the ATC classification level 1, is shown in Table 3. For all countries, antibacterials for systemic use (J01) were the most frequently prescribed, making up 83–99% of prescriptions. Additionally, in Ghana, Uganda, and Zambia, antiprotozoals used as antibacterials (P01AB) made up 4–10% of the prescriptions. Uganda had the highest proportion of prescriptions for antimalarials (P01B) at 4.6% of the total and drugs for treatment of tuberculosis (J04A) at 4.5% of the total. Rates for antibiotics used as intestinal anti-infectives (A07AA), antimycotics and antifungals for systemic use (J02; D01BA) and antivirals for systemic use (J05) were low (0–2%) for all four countries.

The most prescribed antimicrobial across Ghanaian hospitals was metronidazole for systemic use (12% of total prescriptions; 288/2435), compared to ceftriaxone which was prescribed most frequently in the three other countries; Uganda 24% (173/710), Zambia 21% (83/402), and Tanzania 32% (94/290). Full details of the proportions of antimicrobials prescribed in each country can be found in the supplementary data (Tables S1 and S2).

### 2.3. Reason for Prescribing Antimicrobials

**Figure 2:**
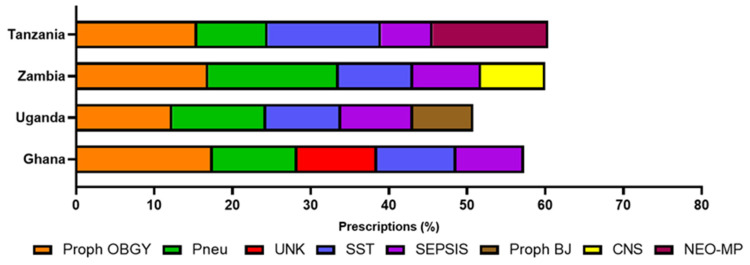
Most common reason for prescribing antimicrobials across 17 hospitals participating in the PPS.

The main reasons for prescribing antimicrobials for each country are shown in Figure 2. Across all countries, antimicrobials were most often prescribed for prophylaxis for obstetric or gynaecological surgery, making up between 12 and 18% of total prescriptions (Figure 2). Antimicrobials were next most frequently prescribed for pneumonia or lower respiratory tract infections, in all but Tanzania, where drugs used as medical prophylaxis for new-born risk factors was the second most common reason for prescribing (15% of total).

In Ghana, the third most common reason for prescribing was to treat skin and soft tissue infections (equal to prescriptions for completely unknown infections). Uganda had a higher prescription rate for prophylaxis for plastic or orthopaedic surgery than other countries (8%) and prescriptions for infections of the central nervous system were higher in Zambia (8%). Drugs were frequently prescribed for sepsis across all countries (7–9%).

Across all four countries, antimicrobials were frequently prescribed for prophylaxis. This practice was highest in Tanzania, with 48% of all prescribed antimicrobials being for prophylaxis. This was followed by in Ghana (33%), Uganda (27%) and Zambia (27%).

Proph OBGY—prophylaxis for obstetric or gynaecological surgery (caesarean section, no episiotomy, carriage of group B streptococcus); pneu—pneumonia or lower respiratory tract infections; UNK—prescriptions for completely unknown infections; SST—skin and soft tissue infections (cellulitis, wound including surgical site infection, deep soft tissue not involving bone, e.g., infected pressure or diabetic ulcer, abscess); proph BJ—prophylaxis for plastic or orthopaedic surgery (bone or joint); CNS—prescriptions for infections of the central nervous system; NEO-MP—medical prophylaxis for new-born risk factors, e.g., VLBW (very low birth weight) and IUGR (intrauterine growth restriction).

Out of 4376 admitted inpatients, 237 patients (5.4%; range 4.1% in Uganda to 5.1% in Zambia) were treated with antibiotics for systemic use (ATC J01) for at least one healthcare associated infection. Table 4 shows the types of indication for which antimicrobials were prescribed by country. Out of all therapeutic prescribing, community-acquired infections were the most common indication for antimicrobial use. Surgical prophylaxis of >1 day (SP3) was common in all countries, up to 97% of all prescriptions for surgical prophylaxis in Tanzania and Uganda (Table 4). Ghana recorded most antimicrobial prescriptions for which the indication was not known (11%).

As mentioned above, one common indication for prescribing antimicrobials in all countries was for prophylaxis for obstetric or gynaecological surgery. The types of antimicrobials prescribed for this indication differed across the four countries. In participating hospitals in Ghana, 16 different drugs were prescribed for OBGY prophylaxis (Table 5), with metronidazole comprising 44% of prescriptions for this indication (186/425). This was split relatively evenly between oral (95/186; 22% of total number of prescriptions) and parenteral administration (91/186; 21.4% of total prescriptions). Of the 186 times metronidazole was prescribed, it was co-administered with another antimicrobial (88%; 164/186). When given orally, it was most commonly prescribed with co-amoxiclav; amoxicillin/clavulanic acid (42/95) and, when given parenterally, it was most commonly prescribed with co-amoxiclav (23/91) or ceftriaxone (22/91).

**Figure 3:**
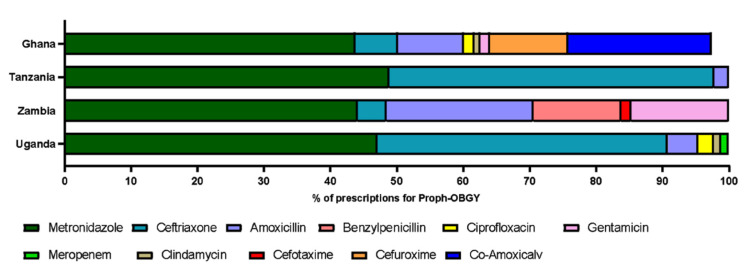
Antimicrobials prescribed for Prophylactic OBGY purposes in four countries (% of total). For Ghana, antimicrobials making up < 1% of prescriptions have been omitted for clarity.

Again, in Ghana, co-amoxiclav was prescribed as a single agent on 19% of occasions (81/425) and, as described, frequently co-administered with metronidazole (65/186). Cefuroxime was the next most frequently prescribed on 12% of occasions (50/425). The top two drugs that were most prescribed in the participating hospitals in Ghana were in the access category, whereas ceftriaxone is in the Watch category. Whilst the most common reason for prescribing antimicrobials was the same for the other 3 countries, there was less variation in drug type (Figure 3).

In Uganda, metronidazole and ceftriaxone were prescribed together frequently for proph OBGY. Metronidazole was prescribed 47.1% of total prescriptions for proph OBGY (41/87) and ceftriaxone 44% (38/87) (Figure 3). They were co-prescribed on 37 occasions, with ceftriaxone only being prescribed alone once for this indication. All but two prescriptions for metronidazole were parenteral administration.

Metronidazole and ceftriaxone were both prescribed for proph OBGY with the same frequency in Tanzania (49%) but were not co-administered on any occasion to the same patient. Metronidazole was also the most prescribed antimicrobial for prophylactic OBGY purposes in Zambia (44%) and was frequently co-administered with amoxicillin (22%) (Figure 3).

### 2.4. Antibiotic Prescription by AWaRe Categories and Age, Gender and Countries

Based on a chi-squared test conducted on the general dataset, there was a significant association between antibiotic prescription across the AWaRe categories and countries (X^2^ = 43.80, *p* < 0.001), gender (X^2^ = 24.81, *p* < 0.001) and age (X^2^ = 97.74, *p* < 0.001). From country-specific analysis, there was a significant association between gender and antibiotic prescription across the AWaRe categories in Ghana (X^2^ = 26.67, *p* < 0.001) and Zambia (X^2^ = 14.12, *p* < 0.001) while the association was not significant in Uganda (X^2^ = 1.46, *p* = 0.482) and Tanzania (X^2^ = 0.41, *p* = 0.815). Furthermore, there was a significant association between age and antibiotic prescription across the AWaRe categories in Ghana (X^2^ = 42.23, *p* < 0.001), Tanzania (X^2^ = 58.50, *p* < 0.001) and Uganda (X^2^ = 22.68, *p* < 0.001). A summary of the statistics is available in Supplementary Tables S3–S5.

### 2.5. Quality Indicators for Prescribing

Quality indicators for all prescriptions were recorded during each survey and include criteria from the GPPS protocol [18] as shown in Table 6. Stop/review dates (i.e., whether a date of review or stop date of the antimicrobial was recorded in the medical records) were almost always reported in both Uganda (99% of prescriptions) and Tanzania (99%). Indications for prescriptions were well-documented ranging from 66% of all prescriptions in Ghana to 97% in Uganda. The proportion of prescriptions that were guideline-compliant varied widely among the four countries from 55% in Zambia, to 88% in Tanzania (Table 6). Ghana most frequently reported the absence of antimicrobial prescription guidelines (13%). Moreover, in Ghana, in 1096 antimicrobial prescriptions (45%), the information with respect to the guidelines was not assessable because the indication was unknown.

### 2.6. Antimicrobial Stewardship Interventions as a Result of PPS

The key antimicrobial stewardship interventions that were implemented as a result of the PPS data in the CwPAMS programme institutions are summarised in Table 7 with further details in SInfo1: Antimicrobial Stewardship Intervention summaries.

## 3. Discussion

The Point Prevalence Surveys presented provide key insights into antibiotic prescribing in selected public or not-for-profit hospitals across Ghana, Tanzania, Uganda, and Zambia. The overall prevalence of antimicrobial use was 50% (30–57%), with most antibiotics prescribed belonging to the WHO ‘Access’ and ‘Watch’ groups. No ‘Reserve’ category antibiotics were prescribed across the study sites; however, there were some prescriptions of ‘Not Recommended’ antibiotic combinations. While this pattern of antibiotic use may partly be due to the prescriber’s consideration of optimality and potential for antimicrobial resistance, in line with WHO’s goal of improving antibiotic use through antimicrobial stewardship [19], other authors suggest alternative reasoning [20,21].

Antibiotic prescribing patterns were significantly associated with accessibility and affordability, with broad-spectrum antibiotics in the Access category being more readily available and affordable than antibiotics in the Watch and Reserve categories and the ‘Not Recommended’ group [20,21]. A recent study on antibiotic availability and use in 20 low- and middle-income countries reported that the median proportion of facilities across countries with availability of Access category antibiotics was 89.5% [22]. Pauwels et al. (2021) reported that low-income countries had the highest percentage of use of Access category antibiotics (63%), the lowest use of Watch category antibiotics (36%) and no Reserve category prescriptions on adult wards across 69 countries [23]. A PPS conducted across six referral hospitals in Tanzania also supports data presented in the current study, reporting 62% of prescriptions for in-patients being from the Access group [24]. In addition to availability and affordability, the similarity in prescription patterns across all four countries in the current study might be associated with the circulating bacterial strains and disease burden across low- and middle-income countries [6,7,25]. The WHO proposes that the AWaRe classification should support monitoring of antibiotic prescribing and inform AMS programmes and has the target that by 2023 at least 60% of national antibiotic consumption should come from the Access category [15]. In data presented here, 60% of the total of ATC J01 antibiotics prescribed in Ghana (1212/2071), 52% in Uganda (309/592), 58% in Zambia (201/347) and 59% in Tanzania (170/288) were in the Access category, demonstrating the hospitals’ alignment with the WHO target for national antibiotic consumption.

The antibiotics most commonly prescribed across hospitals in all four countries in the current study were metronidazole and ceftriaxone, with metronidazole being the most prescribed antibiotic across Ghanaian hospitals. These findings are supported by data from the Pauwels et al. study across 69 countries, where ceftriaxone was the most commonly used antibiotic for therapeutic use on adult wards worldwide. In the same study, up to 24% of prescriptions for surgical prophylaxis in sub-Saharan Africa were for metronidazole, followed by ceftriaxone (23%) [23]. A PPS carried at the Korle-Bu Teaching Hospital in Ghana (2018) reported metronidazole as the most frequently prescribed antibiotic, followed by amoxicillin-clavulanic acid, cephalosporins (ceftriaxone, cefuroxime), and cloxacillin [26]. A PPS in Kenya also recorded a higher use of nitroimidazoles compared to beta-lactam antibiotics [27]. Furthermore, a PPS in three hospitals in north-eastern Tanzania reported ceftriaxone, metronidazole, and penicillin as the most prescribed antibiotics [28].

Metronidazole is effective in the treatment of a broad range of anaerobic infections which may be more common in African countries [29]. Metronidazole was used in addition to co-amoxiclav, so this could perhaps be an area to target to reduce use if anaerobic infections are covered by use of other antibiotics. This highlights that it might be useful to increase the awareness and understanding of antibiotic sensitivity. The findings presented here show that metronidazole was primarily prescribed for prophylaxis for obstetrics and gynaecology. Published data demonstrate a similarity between the most commonly prescribed antibiotics in PPSs carried out in other low-income countries. This could be explained by the affordability, availability, and the spectrum of activity of metronidazole and ceftriaxone, as well as their suitability for prophylaxis in certain obstetric and gynaecological procedures [30]. However, the broad-spectrum activity of some antibiotics, particularly cephalosporins, can lead to the over-growth of other bacteria that are resistant to their activity, for example *Clostridiodies difficile*, methicillin-resistant *Staphylococcus aureus* (MRSA), and vancomycin-resistant enterococci (VRE) [31,32]. The use of cephalosporins, in particular, and third-generation drugs such as ceftriaxone, are linked to the rise in incidence of extended-spectrum beta-lactamase (ESBLs)-producing bacteria, leading to a reduction of effective antibiotics [33,34]. Therefore, there remains a need to evaluate prescriber’s choices and the frequent prescription of ceftriaxone, which falls within the Watch group of antibiotics in the WHO AWaRe categories.

Reasons for antimicrobial prescribing in other published studies demonstrate a similar pattern to the current study, with lower respiratory tract infections and medical and surgical prophylaxis presented as common reasons for prescribing [24,25]. Prophylaxis for obstetric or gynaecological surgery was the most frequent reason for prescribing in the current study. Although the reasons for antimicrobial prescribing were similar across all four countries, Uganda had the highest prescription rates for antimalarials, drugs for the treatment of tuberculosis (TB). The WHO Global Tuberculosis Report (2020) reported Zambia having the highest incidence of TB (of the four countries included here) in 2019 (300–499 incidences per 100,000 population per year), followed by Tanzania (200–299 incidences per 100,000 population per year) and Ghana and Uganda having similar, lower rates (10–99 incidences per 100,000 population per year) [35]. It is possible that there were localised TB and malaria outbreaks in the regions where the Ugandan hospitals were, but data are not available to confirm or deny this. This data may also be influenced by the time of year the PPS was carried out. If the PPS was conducted in the rainy season, then prescriptions for antimalarials might be higher than if the PPS was done in the dry seasons. The reason for this could also be that TB detection methods may be more robust in Uganda, which may explain the lower prevalence of disease but higher use of drugs. Further analysis and PPS would be required to explain the higher observed rates for unclassified antimicrobials and drugs used in the treatment of TB and malaria seen in Uganda. Antimicrobials were often prescribed when indication was documented (Figure 2). There is a need to improve diagnostic capacity as diagnostic uncertainty might lead to the increase of antimicrobial prescribing [36].

From the 4376 admitted inpatients across the 4 countries, 5.4% were treated with antibiotics for systemic use for at least one healthcare-associated infection (HCAI). Out of all therapeutic prescribing, community acquired infections were the most common reason for antimicrobial use. The rates of HCAI were similar to a large study conducted across acute care facilities in 28 countries in the EU/EAA, where 6.5% of patients had at least one HCAI [37]. Although the European study was conducted over a much larger sample group, the data show a trend towards similar rates of HCAI in the data presented in this study.

The chi-squared test of association revealed significant associations between age, gender, countries, and AWaRe categories. Although the exact nature of the relationship between variables is uncertain, the summary statistics (Supplementary Material: Tables S3–S5) provide more insight into these associations. As observed from descriptive analysis, reasons for antibiotic prescribing differ across populations with some age-specific indications such as ‘medical prophylaxis for new-born risk factors’ mostly observed in Tanzania and Uganda and gender-specific indications such as ‘prophylaxis for obstetric or gynaecological surgery’ observed across all countries. While these partly explain significant associations between antibiotic prescribing, gender, and age in specific countries and the general population, our results reflect country-specific trends worthy of further investigation.

Through the Global PPS programme and CwPAMS (for the participating hospitals), all partnerships and additional hospitals in Ghana and Zambia have demonstrated the strengthening of their healthcare workforce knowledge and capacity (Table 7) in the areas of antimicrobial use surveillance and AMS. Prior to the programme, although other hospitals had collated antimicrobial use data through PPS methodology, only one hospital in the four countries had previously conducted data collection to the scale of the Global PPS.

Translating PPS findings into contextualised interventions can be challenging but all CwPAMS health partnerships and hospitals involved have provided information regarding key AMS interventions taken as a result of the Global PPS undertaken at their institutions. These are summarised in Table 7 (for the full text, see Supplementary Data). In an evaluation of the impact of the Global-PPS on local AMS programmes, prolonged surgical antibiotic prophylaxis was the most common target for improvement identified [38]. This study also highlights the need to focus on prolonged surgical antibiotic prophylaxis considering that prescriptions for more than one day for surgical prophylaxis were common. However, antibiotics are only one component that can be used to reduce SSI. The inability to ensure a sterile environment and optimal IPC conditions may result in overuse or antibiotics.

Variations in quality indicators were observed across countries. Consistent with this study, the PPS in 6 reference hospitals in Tanzania observed an 84% adherence of antibiotic prescriptions to the National Standard Treatment Guideline [24]. Similarly, a multi-centre PPS in Ghana also recorded a level of non-compliance to national antimicrobial standard treatment guidelines [39]. The variations observed could be influenced by varying national antimicrobial prescribing policies, hospital protocols and the effectiveness of antimicrobial stewardship intervention programs in study centres across all four countries.

The consistency of data collection via the G-PPS methodology adds rigor and validity to the data presented here. The study demonstrates inclusivity with data obtained from 17 hospitals across four countries in 3 African regions, coupled with a good representation of gender, age groups, and hospital sections. In addition, data were collected by trained health professionals working, for the most part, as part of the Commonwealth Partnerships for Antimicrobial Stewardship.

## 4. Materials and Methods

Training on the collection of surveillance data for the G-PPS reported in this paper was provided to the CwPAMS health partnerships by the Global PPS team and the CPA, supporting the development of evidence-based standards, guidelines, protocols and the development of a mentorship programme to support sustainability. UK volunteers who had experience of PPS also provided mentorship and in-country support during their visits to Ghana, Uganda, Zambia, and Tanzania. Data collection was carried out by volunteers from individual health partnerships. PPS were conducted between May and December 2019, using the G-PPS methodology as described elsewhere [18]. Data from 17 hospitals across four countries: ten in Ghana, four in Uganda, two in Zambia and one in Tanzania were collected and analysed. Follow-up data were collected in a second PPS for two hospitals but only the data from the first survey are included here.

Age of patients were defined as: adult (≥18 years) Child (>1 month–≤17 years) or neonate (≤1 month). Gender, age, diagnosis (reason for prescribing), indication (therapeutic versus prophylactic prescribing), routes of administration, prescribed antimicrobials dosing regimen, and causative microorganisms were all recorded. Data collection also included a set of prescription-related quality indicators; Prescriptions with a documented stop/review date, prescriptions with a documented indication, prescriptions that were guideline compliant and prescriptions for which no guidelines were available. The G-PPS data collection form is available via: https://www.global-pps.com/documents/ (accessed on 15 September 2021).

Hospitals were classified as primary, secondary, or tertiary care hospitals. All inpatients admitted at 8 a.m. on the day of the PPS were included and data were analysed by country and ward type. The included wards were neonatal medical and intensive-care units, paediatric medical, surgical or haematology-oncology ward and intensive-care units and adult medical, pneumology, surgical, haematology-oncology wards, and intensive-care units.

Prescribed antimicrobials were divided into four main categories using the WHO AWaRe classification [14] and further grouped using the 2021 WHO ATC code classification system [20]. AWaRe groups were Access, Watch, Reserve and Not Recommended. Those that were not included in the classification were recorded as unclassified. Antimicrobials were grouped into therapeutic subgroups (ATC 2 level) following the WHO ATC classification system [20]. The therapeutic subgroups were antibacterials for systemic use (J01), antimycotics and antifungals for systemic use (J02 and D01BA including griseofulvin and terbinafine), drugs for treatment of tuberculosis (J04A), antibiotics used as intestinal anti-infectives (A07AA), antiprotozoals used as antibacterial agents, nitroimidazole derivatives (P01AB), antivirals for systemic use (J05) and antimalarials (P01B).

Antimicrobial stewardship interventions as a result of PPS data: leads of all hospitals were asked to provide a short summary of AMS interventions, detailing the actions taken and any follow up data as a result of the PPS.

Data analysis: The results were analyzed descriptively and analytically using the R software (version 4.1.0) and Microsoft Excel (2016). Antimicrobial use prevalence rates were reported by calculating the number of patients on at least one antimicrobial relative to the number of admitted patients at the time of the PPS using Microsoft Excel (2016). The chi-squared test of association was conducted to compare national data on antimicrobial use and identify associations between dependent and independent variables within the dataset using the R software. The tests investigated the association between countries, gender, age groups, and antibiotic prescription across the AWaRe categories. Age was split into three categories namely: neonates (>1 month), children (1 month–17 years), and adults (18 years and above). Statistical significance was set at *p* < 0.05.

Ethics: Formal ethics approval was not required at any hospital as there was no direct patient contact and all data were anonymized. All sites obtained approval from their respective hospital administration. Ethics review and approval was sought and obtained in Uganda and administrative clearance by the participating hospitals was also given. For the four additional sites in Ghana, formal ethical approval was received.

## 5. Conclusions

The prevalence of antimicrobial use in the hospitals included in this study was 50% (30–57%), with most antibiotics prescribed belonging to the WHO ‘Access’ and ‘Watch’ groups. No ‘Reserve’ category antibiotics were prescribed across the study sites. Not Recommended antibiotics were prescribed, albeit infrequently. This aligns with previously published data in that the ‘Access’ and ‘Watch’ category antibiotics are commonly prescribed in LMICs, although to varying extents across countries.

As a result of the CwPAMS health partnership programme and collaboration with other hospitals, PPS data are available for the most part for the first time, strengthening the global commitment to improved antimicrobial surveillance. AMS interventions as a result of the PPSs conducted include formation of AMS committees, preparation of new AMS guidelines and provision of training. Other common interventions included the presentation of findings to clinicians, thus supporting awareness and the multi-disciplinary approach to successful AMS programmes.

In order to continue to monitor the impact of interventions, repeat PPS should be carried out and to strengthen the quality of data, widening participation is encouraged.

## Figures and Tables

**Table 1:** Characteristics of wards and patients (treated with at least one antimicrobial) in PPS conducted at 17 hospitals.

Characteristics	Ghana	Uganda	Zambia	Tanzania
**Number of Hospitals**	10	4	2	1
**Number of Treated Patients**	1366	386	238	179
**Patient Age**				
Neonate (≤1 month)	150 (11%)	20 (5%)	32 (13%)	34 (19%)
Child (>1 month–≤17 years)	261 (19%)	136 (35%)	35 (15%)	29 (16%)
Adult (≥18 years)	955 (70%)	214 (56%)	171 (72%)	116 (65%)
Unknown	-	16 (4%)	-	-
**Patient Gender**				
Male	570 (41.7%)	162 (42%)	96 (40%)	93 (52%)
Female	794 (58.1%)	205 (53%)	142 (60%)	84 (46.9%)
Unknown	2 (0.2%)	19 (5%)	-	2 (1.1%)
**Ward Activity**				
Medicine	76 (46%)	28 (54%)	9 (45%)	7 (35%)
Surgery	74 (45%)	22 (42%)	7 (35%)	10 (50%)
Intensive Care	15 (9%)	2 (4%)	4 (20%)	3 (15%)

**Table 2:** Prevalence of antimicrobial use in four countries included in PPS.

Country	Admitted Patients	Treated Patients	Prevalence of AMU (%)
Ghana	2502	1366	55
Uganda	862	386	45
Zambia	418	238	57
Tanzania	594	179	30
Total	4376	2169	50

**Table 3:** Proportional antimicrobial use by country grouped by therapeutic subgroup.

Therapeutic Subgroup	Proportional Antimicrobial Use (% of Prescriptions)			
	Ghana	Uganda	Zambia	Tanzania
Antibacterials for systemic use (J01)	85.1	83.4	86.3	99.3
Antimycotics and antifungals for systemic use (J02; D01BA)	0.8	1.3	2.2	0.3
Drugs for treatment of tuberculosis (J04A)	1.2	4.5	0.2	0
Antibiotics used as intestinal anti-infectives (A07AA)	0.5	0.3	0.2	0
Antiprotozoals used as antibacterial agents (P01AB)	8.3	4.2	10.2	0.3
Antivirals for systemic use (J05)	1.4	1.7	0	0
Antimalarials (P01B)	2.8	4.6	0.7	0

**Table 4:** Type of indication for antimicrobial prescribing.

	Ghana	Tanzania	Uganda	Zambia
**Total Number of Prescriptions**	2435	290	710	402
**Therapeutic use**	1344 (55.2%)	134 (46.2%)	477 (67.2%)	288 (71.6%)
Community-Acquired infection; CAI	1074 (79.9%)	89 (66.4%)	416 (87.2%)	257 (89.2%)
Healthcare-Associated Infection; HAI	270 (20.1%)	45 (33.6%)	61 (12.8%)	31 (10.8%)
**Prophylactic use**	805 (33.1%)	145 (50.7%)	225 (31.7%)	102 (25.4%)
Medical Prophylaxis; MP	172 (7%)	46 (16%)	50 (7%)	17 (4%)
Surgical Prophylaxis; SP	633 (26.0%)	99 (34.1%)	175 (24.6%)	85 (21.1%)
Surgical Prophylaxis One dose; SP1	42 (6.6%)	3 (3.0%)	2 (1.1%)	1 (1.1%)
Surgical Prophylaxis One day; SP2	113 (17.9%)	0 (0%)	3 (1.7%)	2 (2.3%)
Surgical Prophylaxis > 1 day; SP3	478 (75.5%)	96 (97.0%)	170 (97.1%)	83 (96.5%)
Other (OTH)	19 (0.7%)	1 (0.3%)	1 (0.1%)	8 (2%)
Unknown (UNK)	267 (11%)	10 (3%)	7 (1%)	4 (1%)

**Table 5:** Antimicrobials prescribed for prophylactic OBGY purposes in Ghana (% of total) and AWaRe group.

ATC Code	Drug	% of Prescriptions for Proph-OBGY	AWaRe Classification
P01AB01	Oral Metronidazole	22.4	Access
J01XD01	Parenteral Metronidazole	21.4	Access
J01CF02	Co-amoxiclav/Amoxicillin and enzyme inhibitor	21.6	Access
J01DC02	Cefuroxime	11.8	Watch
J01CA04	Amoxicillin	9.9	Access
J01DD04	Ceftriaxone	6.4	Watch
J01MA02	Ciprofloxacin	1.6	Watch
J01GB03	Gentamicin	1.4	Access
J01FF01	Clindamycin	1	Access
J01FA10	Azithromycin	0.5	Watch
J01AA02	Doxycycline	0.5	Access
J01MA12	Levofloxacin	0.5	Watch
P01AB07	Secnidazole	0.5	Unclassified
J01DD13	Cefpodoxime	0.2	Watch
J01CF05	Flucloxacillin	0.2	Access
J02AC01	Fluconazole	0.2	Unclassified

**Table 6:** Proportion of total prescriptions of all antimicrobials for each of the 4 quality indicators (% of total).

Quality Indicator	Ghana	Uganda	Zambia	Tanzania
Total number of prescriptions	2435	710	402	290
Prescriptions with a documented stop/review date	1579 (67%)	700 (99%)	82 (20%)	289 (99%)
Prescriptions with a documented indication	1601 (66%)	687 (97%)	321 (80%)	248 (86%)
Prescriptions that were guideline-compliant *	858 (84%)	413 (67%)	195 (55%)	237 (88%)
Prescriptions for which no guidelines were available (NA)	313 (13%)	36 (5%)	38 (10%)	1 (0.3%)

* Guideline compliance is calculated as compliance to guidelines (Yes) when guidelines are existing (Yes + No).

**Table 7:** AMS interventions at 16 of the 17 hospitals in response to the PPS carried out.

	Hospital															
AMS Intervention	1	2	3	4	5	6	7	8	9	10	11	12	13	14	15	16
New guidelines developed	X	X		X	X	X	X	X		X	X			X		
Catalyst for forming AMS committee	X	X	X	X					X	X	X	X	X	X	X	X
Improved access to guidelines through:																
Posters	X	X	X													
Printed copies		X	X								X					X
Promotion of CwPAMS app	X	X		X					X		X			X		
Other: Please state												X				
Training sessions	X	X	X	X	X	X	X	X	X	X	X	X	X	X	X	
Repeat PPS conducted and submitted G-PPS platform	X		X	X										X		
Repeat PPS conducted and analysed internally	X	X	X	X					X		X			X		X
AMS awareness raising	X	X	X	X					X	X	X	X		X		
Data presented to clinicians	X	X	X	X	X	X	X	X	X	X				X		
Data presented to AMS committee or DTC	X	X	X	X					X		X			X	X	
Activities for WAAW	X	X	X	X					X					X		
Antibiogram developed			X													
Drug chart updated or created		X											X			X
Improved laboratory services			X		X	X	X	X			X			X		
Other: Please list below																
Quality improvement project on prescriber’s adherence to antibiotics for ambulatory pneumonia management Outpatient A/B prescribing survey		X	X													
Engagement with external stakeholders			X								X					

No details provided by hospital 17.

## Data Availability

Not applicable.

## Supplementary Materials

The following are available online at https://www.mdpi.com/article/10.3390/antibiotics10091122/s1, Table S1: Most common reason for antimicrobial prescribing across 17 hospitals participating in the PPS, Table S2: Proportional use of ATC level 5-defined antimicrobials in each of 4 countries, Table S3: Countries and Antibiotic Prescription by AWaRe Categories, Table S4: Gender and Antibiotic Prescription by AWaRe Categories, Table S5: Age and Antibiotic Prescription by AWaRe Categories. SInfo1: Antimicrobial Stewardship Intervention summaries.
