# Supplementary material for: Antibiotic Prescribing Patterns in Ghana, Uganda, Zambia and Tanzania Hospitals: Results from the Global Point Prevalence Survey (G-PPS) on Antimicrobial Use and Stewardship Interventions Implemented
Source: Antibiotics (Basel). 2021 Sep 17;10(9):1122. doi: 10.3390/antibiotics10091122 (PMC8469030; doi:10.3390/antibiotics10091122)
Supplement: Supplementary file 1 [file antibiotics-10-01122-s001.zip › antibiotics-1332634-SI.pdf]

Supplementary Materials:

Table S1. Most common reason for antimicrobial prescribing across 17 hospitals participating in the PPS.

Country	Reason for prescription				
**Ghana**	Proph OBGY 18%	Pneu 11%	UNK 11%	SST 10%	SEPSIS 9%
**Uganda**	Proph OBGY 12%	Pneu 12%	SST 10%	SEPSIS 9%	Proph BJ 8%
**Zambia**	Proph OBGY 17%	Pneu 17%	SST 9%	SEPSIS 9%	CNS 8%
**Tanzania**	Proph OBGY 15.5%	NEO-MP 15%	SST 14%	Pneu 9%	SEPSIS 6.5%

Table S2. Proportional use of ATC level 5-defined antimicrobials in each of 4 countries.

		Prescriptions (%)			
**Antimicrobial**	**ATC 5 code**	**Ghana**	**Uganda**	**Zambia**	**Tanzania**
Nystatin	A07AA02	0.5	0.1	0.2	0.0
Rifaximin	A07AA11	0.0	0.1	0.0	0.0
Miconazole	A07AC01	0.0	0.0	0.0	0.0
Griseofulvin	D01BA01	0.0	0.0	0.0	0.0
Doxycycline	J01AA02	0.5	0.0	0.5	0.7
Chloramphenicol	J01BA01	0.0	0.0	0.5	1.4
Ampicillin	J01CA01	2.8	7.5	0.0	12.7
Amoxicillin	J01CA04	5.1	2.1	9.0	2.7
Piperacillin	J01CA12	0.0	0.0	0.0	0.7
Benzylpenicillin	J01CE01	1.8	1.1	10.0	1.0
Phenoxymethylpenicillin	J01CE02	0.3	0.6	0.0	0.0
Benzathine benzylpenicillin	J01CE08	0.0	0.0	0.5	0.0
Cloxacillin	J01CF02	1.9	2.8	11.4	1.0
Flucloxacillin	J01CF05	1.9	0.7	0.0	0.3
Co-amoxiclav	J01CR02	9.9	0.0	0.0	1.0
Ampiclox	J01CR50	0.0	6.6	0.0	3.1
Cefalexin	J01DB01	0.0	0.0	0.5	0.0
Cefuroxime	J01DC02	9.9	0.1	0.0	0.0
Cefotaxime	J01DD01	1.6	1.8	2.7	0.0
Ceftazidime	J01DD02	0.2	0.0	0.0	0.0
Ceftriaxone	J01DD04	10.8	24.4	20.6	32.3
Cefixime	J01DD08	0.4	0.3	0.0	0.0
Cefpodoxime	J01DD13	0.3	0.0	0.0	0.0
Ceftriaxone, combinations	J01DD54	0.1	0.0	0.0	0.0
Ceftriaxone and beta-lactamase inhibitor	J01DD63	0.0	0.0	0.0	0.0
Meropenem	J01DH02	1.0	0.6	2.0	0.0
Combinations with long-acting sulfonamides	J01ED20	0.2	0.0	0.0	0.0
Co-trimoxazole	J01EE01	1.5	0.1	2.0	0.7
Sulfamerazine and trimethoprim	J01EE07	0.0	1.0	0.0	0.0
Erythromycin	J01FA01	0.3	0.1	1.2	0.7
Clarithromycin	J01FA09	0.3	0.0	0.0	0.3
Azithromycin	J01FA10	2.7	1.1	1.7	0.7
Clindamycin	J01FF01	6.0	0.3	0.2	0.3

Gentamicin	J01GB03	4.4	8.6	8.5	15.5
Amikacin	J01GB06	1.6	1.3	0.0	0.0
Ciprofloxacin	J01MA02	6.2	3.7	6.7	2.4
Levofloxacin	J01MA12	0.9	0.0	0.0	0.0
Gemifloxacin	J01MA15	0.0	0.0	0.0	0.0
Nalidixic acid	J01MB02	0.0	0.0	0.7	0.0
Penicillins, combinations with other antibacterials	J01RA01	0.0	0.0	0.0	0.0
Ciprofloxacin and metronidazole	J01RA10	0.0	0.0	0.0	0.0
Vancomycin	J01XA01	0.1	0.1	0.5	0.3
Metronidazole	J01XD01	11.8	17.5	7.0	21.0
Nitrofurantoin	J01XE01	0.1	0.0	0.0	0.0
Amphotericin b	J02AA01	0.0	0.1	0.7	0.0
Fluconazole	J02AC01	0.7	1.1	1.5	0.3
Itraconazole	J02AC02	0.0	0.0	0.0	0.0
Rifampicin	J04AB02	0.2	0.6	0.2	0.3
Isoniazid	J04AC01	0.2	0.6	0.0	0.0
Pyrazinamide	J04AK01	0.2	0.6	0.0	0.0
Ethambutol	J04AK02	0.2	1.1	0.0	0.0
Rifampicin and isoniazid	J04AM02	0.1	0.0	0.0	0.0
Rifampicin, pyrazinamide and isoniazid	J04AM05	0.0	0.6	0.0	0.0
Rifampicin, pyrazinamide, ethambutol and isoniazid	J04AM06	0.4	1.1	0.0	0.0
Aciclovir	J05AB01	0.1	0.0	0.0	0.0
Zidovudine	J05AF01	0.0	0.0	0.0	0.0
Lamivudine	J05AF05	0.2	0.0	0.0	0.0
Abacavir	J05AF06	0.0	0.0	0.0	0.0
Tenofovir disoproxil	J05AF07	0.2	0.1	0.0	0.0
Celvudine	J05AF12	0.0	0.1	0.0	0.0
Tenofovir alafenamide	J05AF13	0.0	0.1	0.0	0.0
Nevirapine	J05AG01	0.1	0.1	0.0	0.0
Efavirenz	J05AG03	0.2	0.1	0.0	0.0
Zidovudine, lamivudine and nevirapine	J05AR05	0.0	0.0	0.0	0.0
Lamivudine, tenofovir disoproxil and efavirenz	J05AR11	0.3	0.0	0.0	0.0
lamivudine and tenofovir disoproxil	J05AR12	0.1	0.6	0.0	0.0
lamivudine, abacavir and dolutegravir	J05AR13	0.0	0.0	0.0	0.0
Dolutegravir	J05AX12	0.0	0.6	0.0	0.0
Metronidazole	P01AB01	8.2	4.2	10.2	0.3
Secnidazole	P01AB07	0.1	0.0	0.0	0.0
Proguanil	P01BB01	0.0	0.0	0.0	0.0
Pyrimethamine	P01BD01	0.2	0.0	0.0	0.0
Artemether	P01BE02	0.0	0.0	0.0	0.0
Artesunate	P01BE03	1.2	2.0	0.2	0.0
Artemether and lumefantrine	P01BF01	1.4	2.7	0.2	0.0

Table S3. Countries and Antibiotic Prescription by AWaRe Categories.

	Count Column Percentage (%)			
**Country**	**Access**	**Watch**	**Unclassified**	**Total**
		**General Population**		
Ghana	1439<br>60.0%	883<br>60.3%	164<br>56.6%	1439
Tanzania	334<br>13.9%	202<br>13.8%	21<br>7.2%	557
Uganda	386<br>16.1%	237<br>16.2%	87<br>30.0%	710
Zambia	241<br>10.0%	143<br>9.8%	18<br>6.2%	402
Total	2400<br>57.8%	1465<br>35.3%	290<br>7.0%	4155

Table S4. Gender and Antibiotic Prescription by AWaRe Categories.

	Count Column Percentage (%)			
**Gender**	**Access**	**Watch**	**Unclassified**	**Total**
		**General Population**		
Female	1448<br>60.8%	771<br>53.1%	166<br>57.2%	2385
Male	932<br>39.1%	679<br>46.8%	124<br>42.8%	1735
Total	2380<br>57.8%	1450<br>35.2%	290<br>7.0%	4120
		**Ghana**		
Female	924<br>64.4%	472<br>53.6%	99<br>60.4%	1495
Male	511<br>35.6%	409<br>46.4%	65<br>39.6%	985
Total	1435<br>57.9%	881<br>35.5%	164<br>6.6%	2480
		**Tanzania**		
Female	154<br>46.5%	98<br>48.5%	11<br>52.4%	263
Male	177<br>53.5%	104<br>51.5%	10<br>47.6%	291
Total	331<br>59.7%	202<br>36.5%	21<br>3.8%	554
		**Uganda**		
Female	207<br>55.5%	132<br>58.9%	45<br>51.7%	384
Male	166<br>44.5%	92<br>41.1%	42<br>48.3%	300
Total	373<br>54.5%	224<br>32.7%	87<br>12.7%	684
		**Zambia**		
Female	163<br>67.6%	69<br>48.3%	11<br>61.1%	243
Male	78<br>32.4%	74<br>51.7%	7<br>38.9%	159

Total	241<br>60.0%	143<br>35.6%	18<br>4.5%	402

Table S5. Age and Antibiotic Prescription by AWaRe Categories.

Count Column Percentage (%)				
**Age**	**Access**	**Watch**	**Unclassified**	**Total**
**General Population**				
Neonate	535<br>22.5%	173<br>11.8%	21<br>7.2%	729
Child	322<br>13.5%	234<br>16.0%	60<br>20.7%	616
Adult	1524<br>64.0%	1054<br>72.1%	209<br>72.1%	2787
Total	2381<br>57.6%	1461<br>35.4%	290<br>7.0%	4132
**Ghana**				
Neonate	297<br>20.6%	108<br>12.2%	13<br>7.9%	418
Child	161<br>11.2%	137<br>15.5%	25<br>15.2%	323
Adult	981<br>68.2%	638<br>72.3%	126<br>76.8%	1745
Total	1439<br>57.9%	883<br>35.5%	164<br>6.6%	2486
**Tanzania**				
Neonate	119<br>35.6%	19<br>9.4%	1<br>4.8%	139
Child	49<br>14.7%	24<br>11.9%	2<br>9.5%	75
Adult	166<br>49.7%	159<br>78.7%	18<br>85.7%	343
Total	334<br>60.0%	202<br>36.3%	21<br>3.8%	557
**Uganda**				
Neonate	76<br>20.7%	21<br>9.0%	7<br>8.0%	104
Child	87<br>23.7%	59<br>25.3%	31<br>35.6%	177
Adult	204<br>55.6%	153<br>65.7%	49<br>56.3%	406
Total	367<br>53.4%	233<br>33.9%	87<br>12.7%	687

Antimicrobial Stewardship Intervention summaries

Hospitals included in the PPS data collection were asked to provide feedback regarding interventions implemented as a result of the PPS. These are reported below.

Hospitals 1 & 2

The PPS identified two key quality indicator gaps including missing guidelines for the management of some medical and surgical conditions and poor compliance to empirical antimicrobial policy. The findings informed the content for training sessions and a key part of which was demonstrating and practising the use of the CPA Microguide app to access the Standard Treatment Guidelines

(STG). Local guidelines based on the STG in poster format were developed in collaboration with clinical teams for display in the wards and departments. Compliance with guidelines was increased in both hospitals to reduce the use of broad-spectrum antibiotics (WHO Watch and Reserve antibiotics). Training sessions included an interactive skills practise based on querying non-compliance with guidelines with colleagues to support behaviour change. The project led to a 41% increase in policy compliance and a 19% reduction in the use of co-amoxiclav as first-line treatment (which was against policy). Other notable achievements include improved teamwork and communication between prescribers and pharmacists. Teams in both hospitals have also completed a second PPS and collected data via audits in specific wards to improve prescribing practice. Further analysis of PPS data and additional audits on prescribing for dental conditions are underway as part of a mixed methods study on dental stewardship.

Hospital 3

A second PPS was conducted at the same hospital. Between the 2 survey points, there was an overall reduction in antibiotic prescribing, especially broad-spectrum antibiotics (but these are small numbers and should be interpreted with caution) and there was an improvement in guideline compliance and documentation of review date. There is now a baseline from which the hospital's AMS committee can AMS quality improvements.

Hospital 4

For this partnership, the global PPS was completed on two occasions. The first data collection in July 2019 established the baseline and acted as a focus for engaging and enabling clinical staff in the hospital in AMS activities. The initiative raised awareness of AMS throughout the hospital, as the data collectors were wearing specially commissioned t-shirts, which had key AMS messages (such as "preserve the power of antibiotics" and "handle antibiotics with care – your role matters"). The results were shared with hospital management to support the case for an AMS committee and with clinicians to identify areas for improvement. The second survey was in January 2020 to establish any seasonal variations and to inform and support the creation of a local AMS policy and treatment guidelines. The AMS committee plan to continue using the Global PPS to evaluate the impact of any interventions by the AMS committee.

Hospitals 5–8

The PPS conducted in the hospitals raised awareness of antibiotic misuse and abuse. The results and copies of the analysed report were shared with hospital management to support the case for antibiotic stewardship and with clinicians to identify areas for improvement.

Hospital 9

Following the PPS, the nursing staff recognized the importance of clear and complete documentation of medication details in the treatment charts. The nursing staff also proposed strategies to curb the issue of missed doses among the patients. Also, the medicines and therapeutic committee members examined the finding that the majority of the antimicrobial prescription for surgical prophylaxis were for longer than one day and noted that one key strategy was to improve the infection and prevention interventions in theatre including training the responsible staff, developing and implementing simple and concrete standard operating procedures. The hospital and staff embraced the PPS as a tool to audit antimicrobial use and identify issues of intervention.

Hospital 10

The antimicrobial point prevalence survey took place over 3 days in June 2019, missing guidelines were a recurrent theme, affecting 25% of all prescriptions. Thus, improving access to guidelines, as well as targeted education, and tools to improve antimicrobial prescribing were highlighted for intervention.

A reciprocal visit was arranged for September 2019, and a three-person team consisting of a Microbiology Consultant, Antimicrobial pharmacist, and Infection Control Nurse attended to shadow the Infection Control, and antimicrobial stewardship teams at the hospital for one week. During this period both teams had the opportunity to review the results of the PPS in more detail and plan for future activities. In November 2019, a very successful learning event was held coinciding with World Antibiotic Awareness Week. The CwPAMS app was successfully launched, and learning events were held across the institution for health-care workers and patients, as well as local presentation of PPS findings to clinical leads with suggested courses of action.

Findings of the PPS were submitted to ECCMID, and accepted for poster presentation, at the annual conference, 18-21 April 2020, Paris, France. In January 2021, in collaboration with THET and CwPAMS, a highly successful antimicrobial workshop was held for pharmacists interested in specialising in antimicrobial stewardship. This was the first antimicrobial training event of its kind in the

country and brought together 21 pharmacists (12 internal, 9 external) for specialist training in antimicrobial stewardship by a multi-disciplinary panel of experts.

Hospital 11

Two G-PPS audits (April and October 2019) were carried out in all the wards. The findings helped in baseline assessment and establishing the gaps that existed in the medical practice regarding antibiotic prescribing, which guided the AMS training module. Feedback on audit data was presented to hospital senior management and an AMS sub-committee was formed. Treatment guidelines were distributed to clinicians and units within the hospital to improve accessibility and improve the use of guidelines. The hospital acquired a microbiological culture and sensitivity machine to identify bacteria and drug-resistance and local guidelines for the treatment of pneumonia and cellulitis have been established.

Antimicrobial resistance profiles have been generated to inform policymakers, revise guidelines and assist in the procurement of antibiotics in Government hospitals. Quarterly training in diagnostic AMR stewardship was due to start in February 2021. Monthly audits and antimicrobial surveillance continue in the hospital. The CwPAMS Microguide App was launched within the hospital together with an App for reporting adverse drug reactions (ADR). The Ministry of Health will be guided by the antimicrobial resistance profile data generated to revise Clinical Guidelines and other policies regarding antimicrobial use.

Hospital 12

Performing the PPS and focussing on AMS has made us improve on the following:

- Formation of an AMS committee in the hospital
- Prescribers are now more aware of the use of some antimicrobials, although there are still some irrational prescriptions due to a lack of adequate information.
- About 60% of healthcare providers now check the guidelines

We would like to continue strengthening awareness through training as new staff are enrolled in the practice. Also, we would also support continuous data collection using G-PPS to improve irrational prescribing in real-time. Sharing audit reports routinely to help change practices. Strong laboratory services improve on culture and sensitivity testing. AMS should be a driver to stop AMR and should be done frequently to avoid irrational use of antimicrobials.

Hospital 13

The team realised that there was a high utilization of antimicrobials as a result of the PPS. Interventions included the development of an Antimicrobial Stewardship manual for healthcare workers, the development of a new drug chart for inclusion in the Antimicrobial Stewardship manual and the strengthening of Infection Prevention and Control practices. The Antimicrobial Stewardship manual has been approved by the Health Professions Council for Continued Professional Development. The drug chart was modified but due to Covid-19, the implementation has been slow, however it is hoped it will be implemented shortly.

Hospital 14

The PPS was successfully carried out, allowing the country to be added to the G-PPS database and comparisons to be made between other hospitals of the same type as well as data collected within other countries. Certain antimicrobials were identified as being of higher usage in our hospital when compared with similar hospital types and other African hospitals e.g., ceftriaxone and ampicillin. The team focused their interventions on the liberal use of broad-spectrum antibiotics using the data collected to raise awareness of prescribing habits. Along with sharing the PPS results with the senior management board, some additional wider interventions were implemented to support AMS. These included the roll out of an extensive training program to 1056 healthcare professionals and members of the public, an IPC campaign, working with educational bodies to increase AMS content in early years pharmacy, development of clinical pharmacy services within the hospital, the introduction and promotion of antimicrobial guidance, promotion of World Antibiotic Awareness Week 2019.

The PPS was repeated in November 2019. The two antimicrobials identified as targets for intervention (ampicillin and ceftriaxone) in the first survey have had their usage reduced in certain clinical areas e.g., treatment of pneumonia showed reduction to 0% and 45% respectively (previously 20% and 70% respectively). These data represent a small sample and cannot be used to draw definitive conclusions; however the apparent trend is supported by anecdotal reports from the Chief Pharmacist of positive behaviour change within staff towards good AMS activity.

Hospital 15

The first G-PPS undertaken at this hospital found that there was a high proportion of patient notes with a documented indication for the antimicrobials prescribed (98.6%). Reassuringly, there were no instances of antimicrobials being prescribed without guidelines being available, indicating that there are no gaps in the National Clinical Guidelines which are used at the hospital. A duration or review date was evident for all prescriptions. Guideline compliance was high at 89.9%, the main reason for non-compliance was that 30 prescriptions for antibiotic surgical prophylaxis were extended beyond one day which is not in keeping with recommendations. This evidence supports the feedback from the antimicrobial stewardship workshops with healthcare practitioners in September 2019 whereby participants reported extended duration of prophylaxis for caesarean section. This practice was reported as doctors and nurses felt they could not rely on optimal infection prevention and control practices in the operating field and thereafter to reduce the risk of surgical site infections developing.

Hospital 16

The challenges that occurred during the PPS at this hospital included lack of availability of first-line drugs, lack of availability of blood culture bottles, limited collection hours for urine samples in the lab (evenings and weekends), Stat doses given in another hospital before attending this hospital and high rates of antimicrobial resistance. Suggestions for how to implement changes with regards to the challenges and findings of the PPS include production of a dedicated antibiotic drug chart, having a printed copy of the guidelines in every OPD and ward, formation of an Antimicrobial Stewardship Committee, implementation of Infection Control Measures, increased hand hygiene compliance, a repeat PPS to complete the cycle and assess improvement.

Hospital 17

No feedback received.
